# NGF promotes, in an autocrine–paracrine manner, metabolic and anti-inflammatory pathways in human and mouse adipocytes

**DOI:** 10.1210/clinem/dgag017

**Published:** 2026-01-20

**Authors:** Michail Deiktakis, Elias Athanasakis, Ioannis Charalampopoulos, Achille Gravanis, Christos Tsatsanis, Maria Venihaki, Andrew N Margioris, Eirini Dermitzaki

**Affiliations:** Laboratory of Clinical Chemistry, Department of Laboratory Medicine, School of Medicine, University of Crete, Heraklion 71003, Crete, Greece; Department of General Surgery, University Hospital of Heraklion, Heraklion 71110, Crete, Greece; Laboratory of Pharmacology, Department of Basic Sciences, School of Medicine, University of Crete, Heraklion 71003, Crete, Greece; Institute of Molecular Biology and Biotechnology, Foundation for Research and Technology Hellas (FORTH), Heraklion 70013, Crete, Greece; Laboratory of Pharmacology, Department of Basic Sciences, School of Medicine, University of Crete, Heraklion 71003, Crete, Greece; Institute of Molecular Biology and Biotechnology, Foundation for Research and Technology Hellas (FORTH), Heraklion 70013, Crete, Greece; Laboratory of Clinical Chemistry, Department of Laboratory Medicine, School of Medicine, University of Crete, Heraklion 71003, Crete, Greece; Institute of Molecular Biology and Biotechnology, Foundation for Research and Technology Hellas (FORTH), Heraklion 70013, Crete, Greece; Laboratory of Clinical Chemistry, Department of Laboratory Medicine, School of Medicine, University of Crete, Heraklion 71003, Crete, Greece; Laboratory of Clinical Chemistry, Department of Laboratory Medicine, School of Medicine, University of Crete, Heraklion 71003, Crete, Greece; Laboratory of Clinical Chemistry, Department of Laboratory Medicine, School of Medicine, University of Crete, Heraklion 71003, Crete, Greece

**Keywords:** metabolism, inflammation, adipocyte, obesity

## Abstract

**Context:**

Nerve growth factor (NGF) affects the development and survival of sympathetic neurons. NGF is also found in non-neural cell lineages that are implicated in immune-endocrine interactions associated with metabolic diseases. Although NGF is expressed in white adipose tissue (WAT), little is known about the regulation of its expression and role in adipocytes.

**Objective:**

To determine whether NGF and its receptors are expressed in human adipocytes and demonstrate their role in adipocyte metabolic and inflammatory phenotypes.

**Methods:**

The expression of NGF and its receptors, tropomyosin-related kinase A (TrkA) and pan-neurotrophin receptor (p75^NTR^), and their effects on metabolic and inflammatory responses were examined in freshly isolated adipocytes from human abdominal WAT and mouse 3T3L1 cells.

**Results:**

TrkA and p75^NTR^ were expressed in both human and mouse adipocytes and pre-adipocytes. NGF was secreted by human white adipocytes and their exogenous exposure to NGF increased mitochondrial mass and activity, peroxisome proliferator-activated receptor gamma, CCAAT/enhancer binding protein alpha, and adiponectin levels. Additionally, NGF increased lipolysis in human WAT explants and suppressed lipids accumulation, lipoprotein lipase, and pro-inflammatory mediators interleukin (IL)-6 and IL-8 in human white adipocytes. The pro-inflammatory factor lipopolysaccharide downregulated the levels of NGF receptors in human white adipocytes. NGF also affected mitochondrial activity in both human and mouse pre-adipocytes and TrkA appeared to mediate, at least partially, the effects of NGF on adipocytes.

**Conclusion:**

Our data suggest that NGF is produced locally within the adipose tissue where it upregulates mitochondrial function while it suppresses the pro-inflammatory phenotype of human and mouse adipocytes.

Nerve growth factor (NGF) is a neurotrophin essential for the development of sympathetic neurons (1) and exhibits paracrine/autocrine effects in non-neural cells as well. Although NGF is found in human white adipose tissue (WAT) (2), little is known about its expression and role in adipocytes.

White adipocytes are the main population in the WAT depot that stores energy in the form of triglycerides. Chronic energy imbalance by high caloric intake and low energy expenditure initiates changes in white adipocytes (especially those found in the visceral WAT depot) to store excess energy, and white adipocytes eventually obtain a dysfunctional and hyperplastic phenotype with a single oversized lipid droplet in the cytoplasm and few mitochondria. Under these circumstances, pre-adipocytes, the precursor cells of adipocytes, may undergo differentiation to form extra white adipocytes, a process called adipogenesis leading to hypertrophy of WAT. Meanwhile, M1 macrophages are attracted to the WAT depots (3). In general, the metabolic dysfunction and low-grade metabolic inflammation initiated in the visceral WAT are hallmarks of metabolic syndrome: obesity, type 2 diabetes, and atherosclerosis. Elevated plasma levels of NGF have been detected in patients at the early stages of metabolic syndrome (4) while low plasma levels of NGF have been found in cardiometabolic diseases (5) or in later stages of metabolic syndrome. Those results suggest that NGF may play a role in the pathogenesis of metabolic syndrome.

WAT is a source of a plethora of factors (adipokines, cytokines, neuropeptides, neurotrophins, etc.) that may affect the extent of differentiation of pre-adipocytes to white adipocytes and the dominant white adipocyte phenotype. Conversely, all factors that regulate metabolic processes such as lipolysis (the breakdown of triglycerides to produce glycerol and free fatty acids), glycolysis (the breakdown of glucose for lipid synthesis), oxidative phosphorylation (ATP production within mitochondria) or mitochondrial remodeling may further contribute to adipocyte differentiation and function affecting WAT plasticity overall. Such factors like the neuropeptide noradrenaline that reaches tissues through sympathetic innervation (especially in brown adipose tissue [BAT]) increase lipolysis. Beyond the trophic effects of NGF in sympathetic neurons, NGF upregulates glucose-dependent insulin secretion from rat pancreatic beta cells (6) and modulates proliferation and differentiation in human immune cells (B and T lymphocytes (7, 8)), cultured endothelial cells and vascular smooth muscle cells (9).

NGF acts by binding to 2 distinct types of receptors, the high-affinity receptor tropomyosin-related kinase A (TrkA) and the low-affinity pan-neurotrophin receptor (p75^NTR^ (10)). The well-studied mouse pre-adipocyte cell line 3T3L1 expresses the mRNA levels of NGF receptors, TrkA and p75^NTR^, and pro-inflammatory cytokines differentially affect NGF expression pattern in 3T3L1 cells (11). The mRNA levels of NGF are also expressed in murine and human whole adipose tissues (2). During the development of obesity, the production of chemoattractant factors, pro-inflammatory cytokines, and adipokines are all upregulated except for the decrease of adiponectin levels (12). Whether NGF and its receptors are expressed in human white adipocytes and pre-adipocytes and if their expression is controlled by the Gram-negative bacterial lipopolysaccharide (LPS), that is detected in the adipose tissue because of increased intestinal permeability in obesity (13), has not been studied yet. As WAT, in contrast to BAT, exhibits metabolic dysfunction and an enhanced pro-inflammatory phenotype during the development of obesity (14), we examined the presence of NGF and its receptors in human and mouse adipocytes and its possible role in the regulation of the metabolic and inflammatory phenotypes of white adipocytes.

Here, we report that primary human white adipocytes in culture produce NGF, its low-affinity receptor p75^NTR^ is abundantly expressed, and its high-affinity receptor TrkA is also detected in these cells. NGF decreased the accumulation of lipids in human white adipocytes and increased lipolysis in human WAT explants. NGF affected metabolism of human white adipocytes by increasing mitochondrial mass, and induced expression of peroxisome proliferator-activated receptor gamma (PPAR-gamma), CCAAT/enhancer binding protein alpha (CEBPA), and adiponectin. NGF also enhanced mitochondrial activity in both human and mouse pre-adipocytes. Chronic exposure of human pre-adipocytes to NGF maintained their pre-adipocyte phenotype by promoting adiponectin and decreasing expression of the lipid metabolism marker perilipin1 (PLIN1). In parallel, NGF reduced the levels of the chemoattractant interleukin (IL)-8, the cytokine IL-6, and toll-like receptor 4 (TLR4) receptor in human white adipocytes, which was also confirmed in 3T3L1 pre-adipocytes since it reduced the expression of C-X-C motif chemokine ligand 1 (CXCL1), the murine analogue of IL-8. TrkA appeared to mediate, at least partially, the effects of NGF on adipocytes. Lastly, NGF secretion and function were under the control of LPS which is enhanced in obesity.

## Methods

### Ethics statement

The protocol was approved by the Ethics Committee of the University Hospital of Heraklion, Crete (No. 16900/13357) and all participating subjects signed informed consents. The study was conducted as per the principles expressed in the Declaration of Helsinki.

### Reagents and antibodies

Mouse NGF was purchased from Millipore (01-125) (the mature peptide of this protein is identical in mammals including human, pig, rat and mouse) and the TrkA inhibitor from MedChemExpress (GW 441756). Collagenase Type I (17100-017) and all culture media were obtained from Invitrogen (Carlsbad, CA). Bovine serum albumin (BSA), insulin, dexamethasone, sodium bicarbonate, 3-isobutyl-1-methyl-xanthine (IBMX), LPS, and Oil-Red-O were purchased from Sigma. Bradford Coomassie Brilliant Blue G-250 was obtained from Bio-Rad Laboratories (Hercules, CA) and the tissue apparatus was obtained from Corning (Corning, NY). All other chemicals and reagents were obtained from Sigma, if not stated otherwise.

### Isolation of human primary adipocytes

Abdominal adipose tissue biopsies were obtained from patients undergoing general surgery. In total, 20 tissues were donated by 10 male and 10 female patients: age (years) 58.15 ± 13.05, body mass index 28.6 ± 5.8, glucose (mg/dL) 96.7 ± 16.2 (data depicted as mean ± SD). None of the donors exhibited metabolic syndrome. Human primary white adipocytes and stromal vascular fraction (SVF) cells were isolated from tissue biopsies as previously described (15). Following isolation, SVF cells were treated with lysis buffer for the elimination of erythrocytes, and cells were maintained under humidified conditions in an incubator with continuous supply of 5% CO_2_ at 37 °C. The growth medium was changed 3 times a week and after differential plating, cells were ready for experimentation.

### Mitochondria staining and mass measurement

White mature adipocytes were cultured (250 000 cells/well) in basal growth medium. The next day, cells were incubated with fresh growth medium (control group) or growth medium supplemented with NGF or LPS at 100 ng/mL for the indicated time intervals (24 hours, 30 hours). White mature adipocytes were continuously floating during experimentation due to their high contents of lipids. At the appropriate time point, cells were centrifuged and stained with Mitotracker Green FM (Thermo Fischer) in D-PBS buffer containing 0.05% FBS for 30 minutes at 37 °C. At the final step, cell lysates were transferred to a 96-well black plate (Corning; 3603) to measure the optical density (absorbance: 490 nm, emission: 535 nm) in a VICTOR X4 Multimode plate reader (PerkinElmer Inc).

### Biochemical measurements in human adipocytes and 3T3L1 cells

For the determination of NGF secretion from primary human white mature adipocytes, cells were cultured in growth medium (basal levels) or growth medium supplemented with LPS (100 ng/mL) and supernatants were collected 6 hours or 24 hours post-culture and stored at −80 °C for further analysis. The levels of NGF in the supernatants were measured with the Human Adipocyte Magnetic Bead panel kit (Millipore Cat# HADCYMAG-61K, RRID:AB_3720843) and the levels of pro-inflammatory mediators (IL-8, IL-6, tumor necrosis factor alpha [TNFα], IL-1β, interferon gamma [IFNγ], IL-17α) with the Human Cytokine/Chemokine/Growth Factor Magnetic Bead Panel kit (Millipore Cat# HCYTA-60K-PX38, RRID:AB_3720844) as previously described (15).

For the determination of human leptin from primary human white mature adipocytes in culture, supernatants were collected after appropriate experimentation and tested for the detection of leptin using the Human Leptin ELISA Development Kit (ABTS) (Thermo Fisher Scientific Cat# 900-K90, RRID:AB_3720837) according to the manufacturer's instructions.

The 3T3L1 pre-adipocyte cell line (RRID:CVCL_0123) was provided by the American Type Culture Collection. The 3T3L1 pre-adipocytes were plated at a density of 200 000 cells/well overnight and then incubated with growth medium treated with NGF (100 ng/mL) plus/minus TrkA inhibitor (10^−6^ M) for 3 hours. Cell supernatants were collected and stored at −80 °C. The mouse CXCL1/KC DuoSet ELISA kit (R and D Systems Cat# DY453, RRID:AB_3675362) was used for the measurement of CXCL1 in supernatants and data were normalized by the total protein levels of the samples as previously described (16).

### Measurement of intracellular lipid contents

Human white mature adipocytes were cultured in basic growth medium supplemented with NGF (100 ng/mL) or LPS (100 ng/mL) for 24 hours or 30 hours. At the end of the indicated incubation periods, cells were fixed in 3.7% HCHO in PBS and incubated with Oil-Red-O solution for 45 minutes at room temperature, which resulted in the crystallization of intracellular lipid droplets. Then, crystals were dissolved by isopropanol, and the optical density of the samples were measured at 595 nm in a Microplate Reader Model 680 (Bio-Rad, USA). The degree of the differentiation of the cells (accumulation of lipid droplets) was proportional to the optical density.

### Ex vivo lipolysis

Lipolysis of adipose tissue was examined as previously described (17). In brief, isolated adipose tissue samples were weighed (∼160 mg), cut into 6 smaller pieces and incubated in DMEM containing 2% fatty acid-free BSA in the presence or absence of noradrenaline (10^−7^ M), insulin (10nM) or NGF (100 ng/mL) for 2 hours or 24 hours. The supernatants were collected, and fatty acid release was quantified using free fatty acid assay kit (ab65341) in Microplate Reader Model 680 (Bio-Rad, USA). The results were normalized to the weight of each fat pad per sample.

### Measurement of cell mitochondrial activity

To test the effect of different doses of NGF on cell's activity, cells were cultured in growth medium supplemented with NGF (1, 10 or 100 ng/mL) or plain growth medium (control cells) for 1 or 3 days in 96-well plates. At the end of the indicated incubation periods, the dye 3-(4,5-dimethylthiazolyl-2)-2,5-diphenyltetrazolium bromide (MTT) was supplemented for 4 hours at 37 °C. Finally, the optical density of the samples was measured in a spectrometer ELISA (Anthos Reader 2001) at 595 nm and the resulting values were directly proportional to the number of metabolically active cells.

### Isolation of total RNA, cDNA synthesis, and reverse transcription–polymerase chain reaction

Total cellular RNA was extracted from human white mature adipocytes and pre-adipocytes using TRIzol reagent (Invitrogen) as previously described (15). In parallel, in vitro differentiated Thp1 macrophages (treated with LPS plus IFNγ) were used as positive control samples (M1 macrophages) for the expression of TLR4. The cDNA synthesis was performed by reverse transcription (TAKARA BIO INC) using 500 ng of total RNA from each sample which were then amplified (95 °C for 15 seconds, 60 °C for 30 seconds for a total of 40 cycles) using the specific primers (Supplemental Table 1 (18)) by reverse transcription–polymerase chain reaction (RT-PCR) as previously shown (15). GAPDH was used as reference gene and the results were calculated with the ΔΔCt method. For instances of NGF, TrkA, p75^NTR^, TLR4, and GAPDH, the amplified cDNA samples were loaded into a 1.5% agarose gel for electrophoresis and visualized under UV light.

### In vitro differentiation of pre-adipocytes to white adipocytes

Human SVF pre-adipocytes or the 3T3L1 pre-adipocyte cell line were cultured in plain growth medium and then in a cocktail of compounds to induce differentiation (For more information, see Supplementary Methods (18)).

### Measurement of mitochondrial respiration and glycolysis rate

Oxygen consumption rate (OCR) and glycolysis rate (extracellular acidification rate [ECAR]) were measured in 3T3L1 pre-adipocytes using the Seahorse Phenotype kit (103275-100) in an Agilent Seahorse XFp Analyzer. In brief, cells (30 000 cells/well) were treated with NGF (100 ng/mL) plus/minus TrkA inhibitor (10^−6^ M) or plain growth medium (control group) for 3.5 hours before baseline measurements. To simulate mitochondrial stress conditions, cells were simultaneously treated with oligomycin and carbonyl cyanide 4-(trifluoromethoxy)phenylhydrazone (FCCP) to inhibit the mitochondrial ATP synthesis and depolarize the mitochondrial membrane potential, respectively. Results are expressed as OCR (pmol/min), ECAR (mpH/min) and as the % of the baseline levels of each measurement. We further calculated the metabolic potential of the cells as follows; their highest respiratory capability as the percentage increase of stressed OCR over baseline OCR levels and their highest glycolytic capability as the percentage increase of stressed ECAR over baseline ECAR levels.

Additionally, human pre-adipocytes (80 000 cells/well) or 3T3L1 pre-adipocytes (5000 cells/well) were seeded in Agilent Seahorse XF96 plates in their respective growth medium. The next day, the Mito stress test kit (103015-100) was employed as per manufacturer protocol, and cells were exposed to NGF (100 ng/mL) and/or etomoxir (50μM), followed by serial injections of oligomycin (1.5μM), FCCP (6μM), and rotenone/antimycin A (0.5μM). Alternatively, cells were pre-exposed to etomoxir (50μM) for 60 minutes followed by the serial injections of NGF (100 ng/mL), oligomycin, FCCP, and rotenone/antimycin A. Results are expressed as OCR (pmol/min).

### Measurement of glucose uptake

Human SVF pre-adipocytes were seeded in 96-well plates. On the day of the experiment, cells were serum-starved for 8 hours and then incubated with the appropriate buffer containing 2% BSA to starve cells from glucose for 40 minutes. Then, cells were exposed to NGF (100 ng/mL) plus/minus insulin (1μM) and glucose uptake was measured using the glucose uptake assay kit (ab136955) according to the manufacturer's instructions (Abcam).

### Western blotting

Human primary white adipocytes, 3T3L1 pre-adipocytes and 3T3L1 in vitro differentiated-to-white adipocytes were lysed and whole cell lysates were subjected to Western blot analysis against antibodies for TrkA (1:1000) (Millipore Cat# 06-574, RRID:AB_310180), p75^NTR^ (1:1000) (Promega Cat# G3231, RRID:AB_430853) and actin (1:500) (Sigma-Aldrich Cat# A4700, RRID:AB_476730).

HEK293 cells (RRID:CVCL_0045) were used as negative control for the expression of TrkA and p75^NTR^ (19), and CHO cells (RRID:CVCL_0213), a cell line derived from Chinese hamster ovaries, were used for high expression of the transfected proteins TrkA and p75^NTR^.

### Statistical analysis

For the statistical evaluation, data were checked to determine if values were normally distributed using the Shapiro–Wilk test. When normal distribution criteria were met, Student's *t* test was used for the comparison of 2 independent groups and analysis of variance (ANOVA) for 3 or more groups, followed by Tukey's post hoc test to correct for multiple comparisons. Additionally, Welch's *t* test and Welch's ANOVA were employed when the F test revealed significant differences in variance. When values were not normally distributed, the non-parametric test Kruskal–Wallis was used to compare 2 independent groups and the Mann–Whitney test for 3 or more groups, followed by Dunn's post hoc test to correct for multiple comparisons. The alpha level was set at 0.05 for statistically significant differences. Values are expressed as mean ± standard error of the mean (SEM). Data were processed in Microsoft Excel spreadsheet and analyzed using GraphPad Prism 8 (GraphPad Software Inc., San Diego, CA, USA, RRID:SCR_002798).

## Results

### Detection of NGF and its receptors in adipocytes derived from human WAT

**Figure 1:**
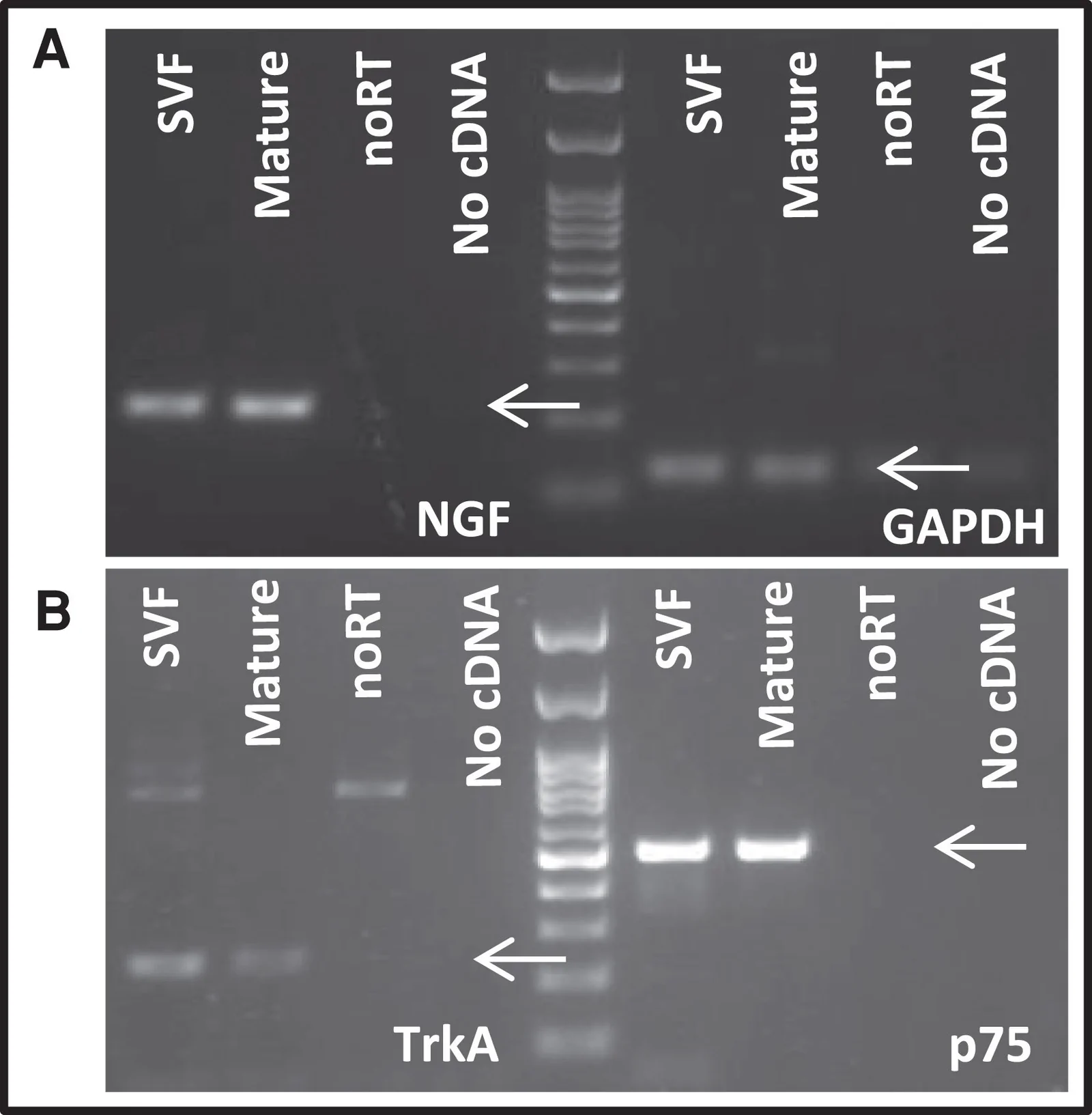
Expression of NGF and its receptors in human WAT. mRNA levels of NGF (Panel A) and receptors TrkA and p75^NTR^ (Panel B) measured by RT-PCR in SVF samples and isolated human primary white adipocytes (Mature). GAPDH was used as a housekeeping gene. Samples without reverse transcriptase during cDNA synthesis (noRT) and without cDNA during RT-PCR (No cDNA) were also shown.

We examined the expression pattern of NGF and its receptors, TrkA and p75^NTR^, in cells from human SVF fraction and isolated human white mature adipocytes. We confirmed the presence of NGF mRNA in human white mature adipocytes, which revealed a higher expression pattern compared to the cells from SVF fraction (Fig. 1A). NGF protein levels were also secreted from isolated human primary white mature adipocytes under basal growth conditions (Supplemental Fig. S1A (18)).

TrkA mRNA was detectable in both SVF and human mature white adipocytes and p75^NTR^ mRNA was highly expressed in both cell types (Fig. 1B). These results were also confirmed by the detection of the protein levels of the receptors in human white mature adipocytes (Supplemental Fig. S1B (18)).

### NGF affected the phenotype of human primary white mature adipocytes

NGF, as a growth factor, regulates metabolism of neural cells and multiple lines of evidence suggest its growth role in non-neural cells as well (7). Thus, we next exposed human white mature adipocytes to NGF in vitro, and measured (a) the amount of their mitochondrial mass and their metabolic activity; (b) the expression levels of transcription factors PPAR-gamma (an important regulator of mitochondria biogenesis and lipids metabolism), and CEBPA and CEBPB, which act in conjunction with PPAR-gamma to promote adipogenesis; and (c) the mRNA levels of adiponectin, a principle adipokine affecting lipid metabolism regulating adipogenesis and glucose metabolism promoting insulin-sensitivity (20).

**Figure 2:**
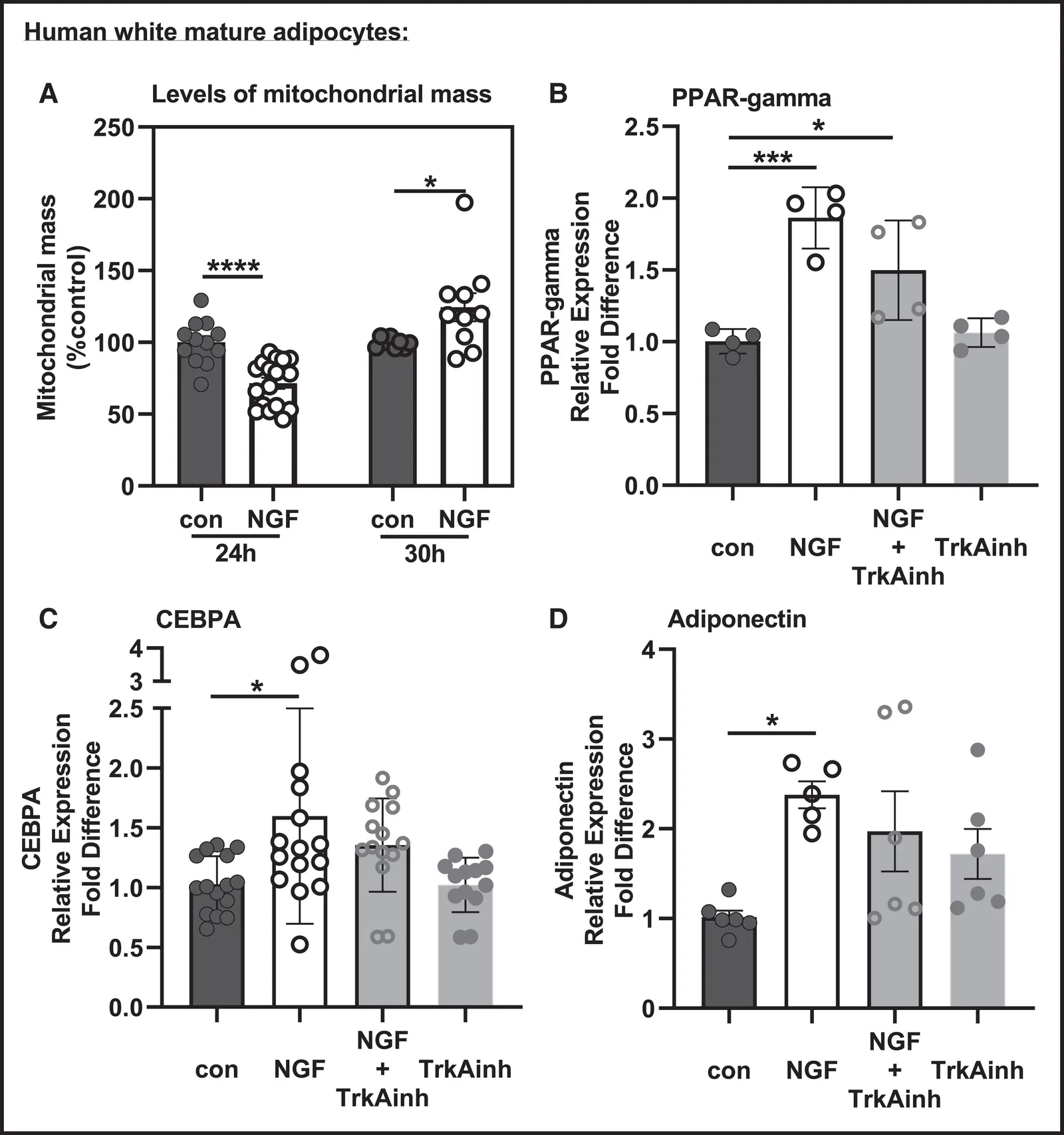
NGF affected the phenotype of human white mature adipocytes. (A) Isolated human white mature adipocytes were cultured in basic growth medium plus/minus NGF (100 ng/mL) for 24 hours or 30 hours and stained with Mitotracker dye to detect the amount of mitochondrial mass. Data are presented as the percentage of mitochondrial mass of the corresponding control cells (mean ± SEM%). Data for 24-hour measurements were employed from 5 independent human biopsies and for 30-hour measurements from 4 independent human biopsies. (B–D) Isolated human white mature adipocytes were cultured in the presence of NGF (100 ng/mL) plus/minus TrkA inhibitor (10^−6^ M) for 24 hours and the mRNA levels of PPAR-gamma (B), CEBPA (C), and adiponectin (D) were measured by RT-PCR. Data are depicted as mean ± SEM and represent the fold difference of the respective mRNA levels measured in the control group. Data for PPAR-gamma mRNA measurements were employed from a human biopsy, for CEBPA mRNA measurements from 3 independent human biopsies, and for adiponectin mRNA measurements from 2 independent human biopsies. *P < .05, ***P < .001 and ****P < .0001 denote statistical significance of the respective levels measured in the control group.

Cells were exposed to different concentrations of NGF (1-1000 ng/mL) and the concentration of 100 ng/mL was chosen as the optimum concentration for consistent results (data not shown). NGF triggered a time-dependent effect on the amount of mitochondrial mass in human white adipocytes. Indeed, 24-hour exposure to NGF reduced the amount of mitochondrial mass (mean ± SEM%: 71.5% ± 3.9%; *P* < .001), which was subsequently increased at 30 hours post-NGF treatment (124.5% ± 9.8%, *P* < .05), all compared to the amount of mitochondrial mass measured in control cells at the indicated time intervals (Fig. 2A).

To elucidate whether the robust decrease in mitochondrial mass of human white mature adipocytes by NGF treatment was not attributed to cell death, we measured their metabolic activity by MTT. Primary white enlarged adipocytes are sensitive to manipulation due to their high content of lipids; thus, we decided not to extend the cultured period for too long. We verified that a 24-hour exposure of human white mature adipocytes to NGF (100 ng/mL) marginally increased their metabolic activity compared to control cells (Supplemental Fig. S2 (18)).

As human white adipocytes seemed to be metabolically active at 24-hour exposure to NGF, we next investigated the metabolic mechanisms activated by NGF that may trigger a subsequent increase in the amount of their mitochondrial mass (at 30-hour treatment with NGF). Therefore, we investigated whether a 24-hour exposure of human primary white adipocytes to NGF affected the mRNA levels of transcription factors PPAR-gamma, CEBPA, and CEBPB. We discovered that a 24-hour treatment of human white mature adipocytes with NGF at 100 ng/mL statistically increased the mRNA levels of PPAR-gamma (mean ± SEM: 1.8 ± 0.1-fold difference, *P* < .001) and of CEBPA (1.6 ± 0.2-fold difference, *P* < .05) compared to the same mRNA levels measured in control groups. Interestingly, these effects were blocked to some extent by the simultaneous addition of a TrkA receptor inhibitor (10^−6^ M) for 24 hours for either PPAR-gamma or CEBPA (Fig. 2B and 2C). NGF did not affect the mRNA levels of CEBPB in human white mature adipocytes (data not shown).

Furthermore, exposure of human white mature adipocytes to NGF (100 ng/mL) for 24 hours caused an increase in the mRNA levels of adiponectin (2.38 ± 0.15-fold difference, *P < .05) compared to the control group (1.01 ± 0.07). The simultaneous exposure to the TrkA inhibitor (10^−6^ M) tended to decrease adiponectin mRNA levels (Fig. 2D). Exposure of human white adipocytes to TrkA inhibitor (10^−6^ M) alone, did not affect PPAR-gamma, CEBPA, or adiponectin mRNA levels in a significant level (Fig. 2B–2D).

### NGF decreased lipid storage in human white mature adipocytes

**Figure 3:**
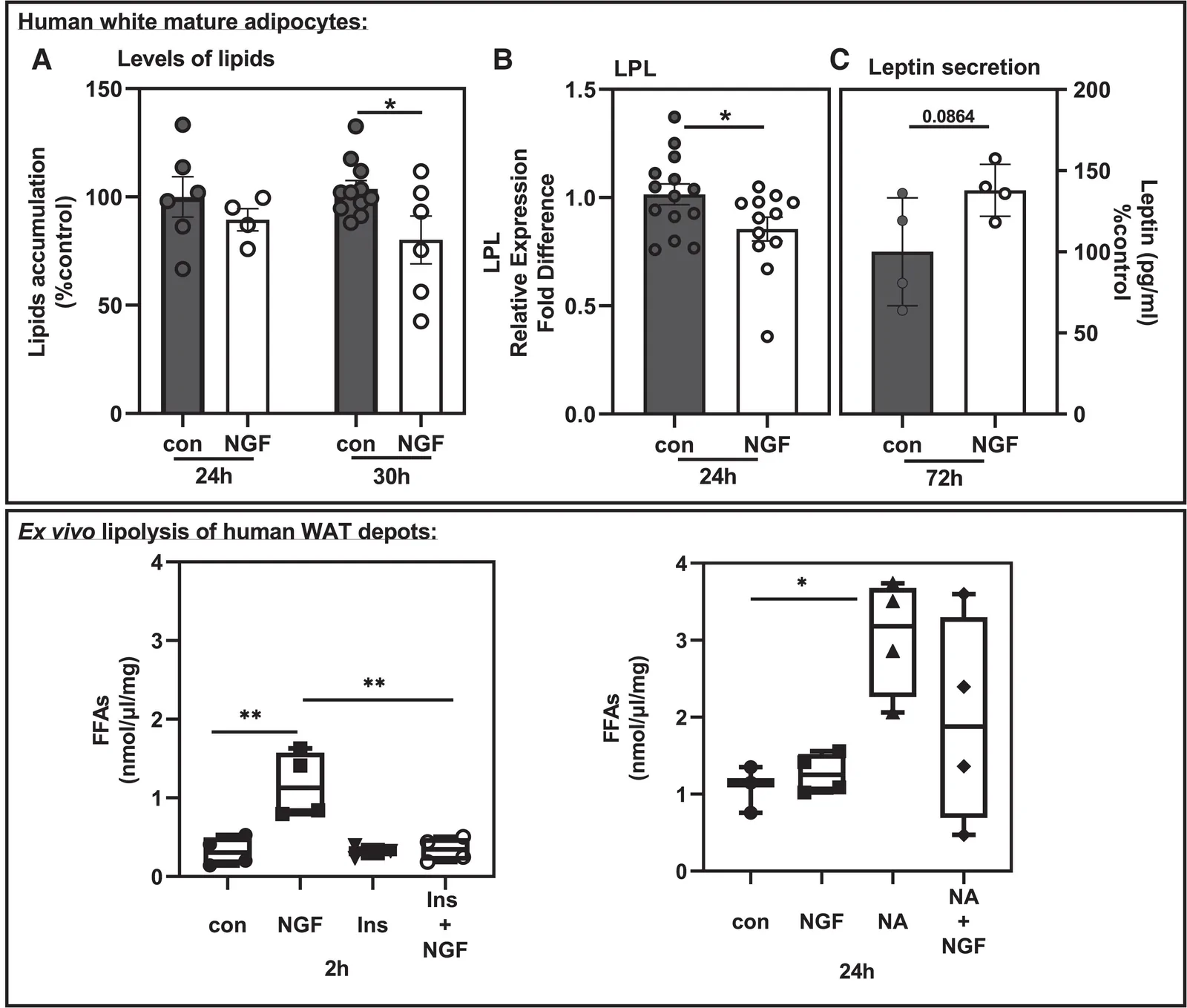
Effect of NGF on lipids accumulation and leptin secretion from human white adipocytes. (A) Isolated human white mature adipocytes were treated with NGF (100 ng/mL) for 24 hours or 30 hours, stained with Oil-Red-O dye, lysed and the amount of lipids was measured as the absorbance read at 595 nm. Data was collected from white mature adipocytes isolated from 5 independent human biopsies. The mRNA levels of LPL were measured upon 24-hour exposure to NGF (100 ng/mL) (B), and the secretion levels of leptin were measured upon 72-hour exposure to NGF (C). Data are presented as the percentage of mean ± SEM measured in the respective control groups for lipids and for leptin secretion levels. Data are presented as mean ± SEM and represent the fold difference of the respective mRNA levels measured in the control group for LPL. Statistically significant signs (*P < .05, **P < .01) depict the comparison of each group of measurements to the relevant control group. Lower panel: Ex vivo lipolysis in human WAT depots. Human visceral WAT depots were exposed to NGF (100 ng/mL), insulin (10nM) or noradrenaline-NA (10^−7^ M) at the indicated time intervals and the release of free fatty acids was measured in the supernatants. Data are presented as mean ± SEM from 2 independent human biopsies.

The increase in lipid accumulation in white adipocytes shapes up their hyperplastic phenotype in obesity. Thus, we examined the impact of NGF on the amount of lipids in human white adipocytes. We found that exposure of human primary white adipocytes to NGF (100 ng/mL) resulted in reduction of the intracellular lipid content compared to the control group, which was significantly evident at 30 hours of exposure (mean ± SEM%: NGF 80.1% ± 11.0% vs control 103.7% ± 3.9%, *P* < .05) (Fig. 3A).

As adipocytes express lipoprotein lipase (LPL), which is the rate-limiting enzyme for fatty acid storage, we measured the mRNA levels of LPL in response to 24-hour treatment of human white mature adipocytes with NGF. We found that NGF significantly decreased the mRNA levels of LPL (*P* < .05) (Fig. 3B). Since leptin levels are increased in obesity, we also measured the secretion of leptin by white mature adipocytes and whether NGF could affect these levels. Leptin secretion was marginally elevated in the supernatant of white mature adipocytes upon 72 hours of treatment with NGF (Fig. 3C).

### Regulation of lipolysis in visceral WAT depot by NGF

Having established the role of NGF on lipid accumulation in human white mature adipocytes in vitro, we next investigated the effect of NGF on lipolysis using whole WAT explant as a model. Lipolysis is the procedure of breakdown of triglycerides to produce glycerol and free fatty acids and is dynamically activated depending on the organism's energy needs. For this experiment, we measured the release of free fatty acids in the supernatant from ex vivo visceral WAT depots. WAT depots were also exposed to insulin that decrease lipolysis (21) and to noradrenaline, which is the potent factor activating lipolysis in BAT. We found that NGF significantly increased free fatty acid release from WAT explants and that this effect was diminished by insulin treatment. Noradrenaline was a potent inducer of ex vivo free fatty acids released from WAT depots (Fig. 3, lower panel).

### Characterization of the pre-adipocyte phenotype of human SVF cells

**Figure 4:**
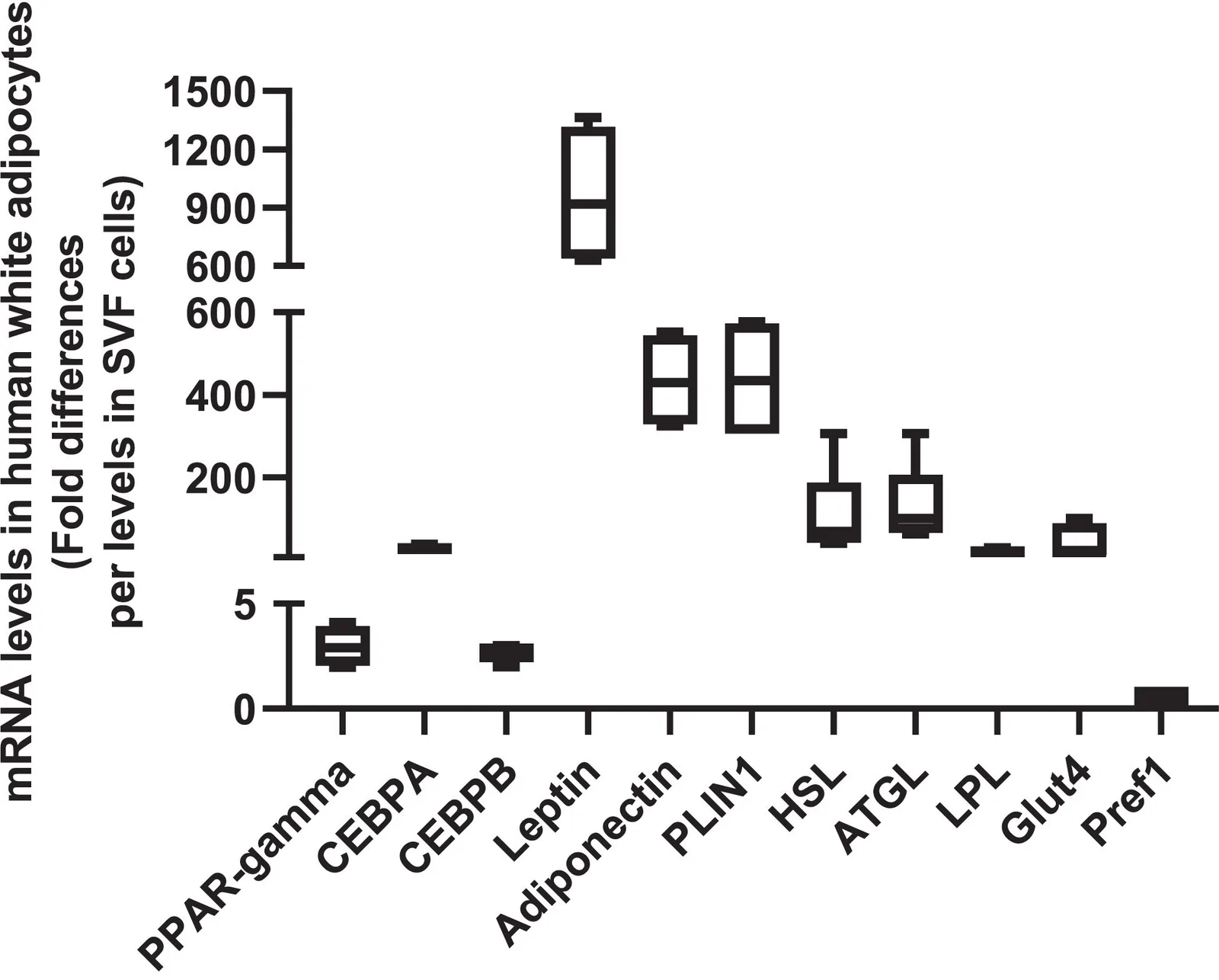
Basal adipogenic phenotype of human white mature adipocytes in comparison to the basal phenotype of human SVF cells. The mRNA levels of PPAR-gamma, CEBPA, CEBPB, leptin, adiponectin, LPL, PLIN1, HSL, ATGL, Glut4, and Pref1 were measured by RT-PCR in isolated human primary white adipocytes and SVF samples. GAPDH was used as a housekeeping gene. Data are depicted as mean ± SEM and represent the fold difference of the respective mRNA levels measured in human SVF cells. Data from 5 independent human biopsies.

Cells isolated after the differential plating of human SVF fraction were analyzed for their adipogenic phenotype in comparison to the adipogenic phenotype of human white mature adipocytes. To characterize the adipogenic phenotype of human white mature adipocytes and human SVF cells, we analyzed the mRNA levels of markers of adipogenesis; transcription factors (PPAR-gamma, CEBPA, CEBPB), adipokines (leptin, adiponectin), lipid metabolism markers (LPL, PLIN1), lipolytic enzymes (hormone-sensitive lipase [HSL], adipose triglyceride lipase [ATGL]), glucose transporter protein (Glut4) in adipocytes, and the pre-adipocyte marker (Pref1) (22, 23). The mRNA levels of all adipogenic markers were detected in SVF cells but at low levels (except for the detection of Pref1 that was higher in SVF cells) all compared to white mature adipocytes. The results are depicted as the fold difference of the mRNA levels detected in human white mature adipocytes compared to SVF cells (Fig. 4). Thus, we conclude that the SVF cells are composed mainly of pre-adipocytes although the existence of small populations of other cell types cannot be totally excluded.

### NGF affected metabolism of human pre-adipocytes and during their in vitro differentiation to white adipocytes

Human pre-adipocytes were exposed to different doses of NGF for 1 to 3 days, and the mitochondrial activity was measured by MTT. The treatment of human pre-adipocytes with different doses of NGF (1, 10, or 100 ng/mL) for 1 to 3 days, led to an initial decrease followed by an increase in their mitochondrial activity (Supplemental Fig. S3 (18)).

**Figure 5:**
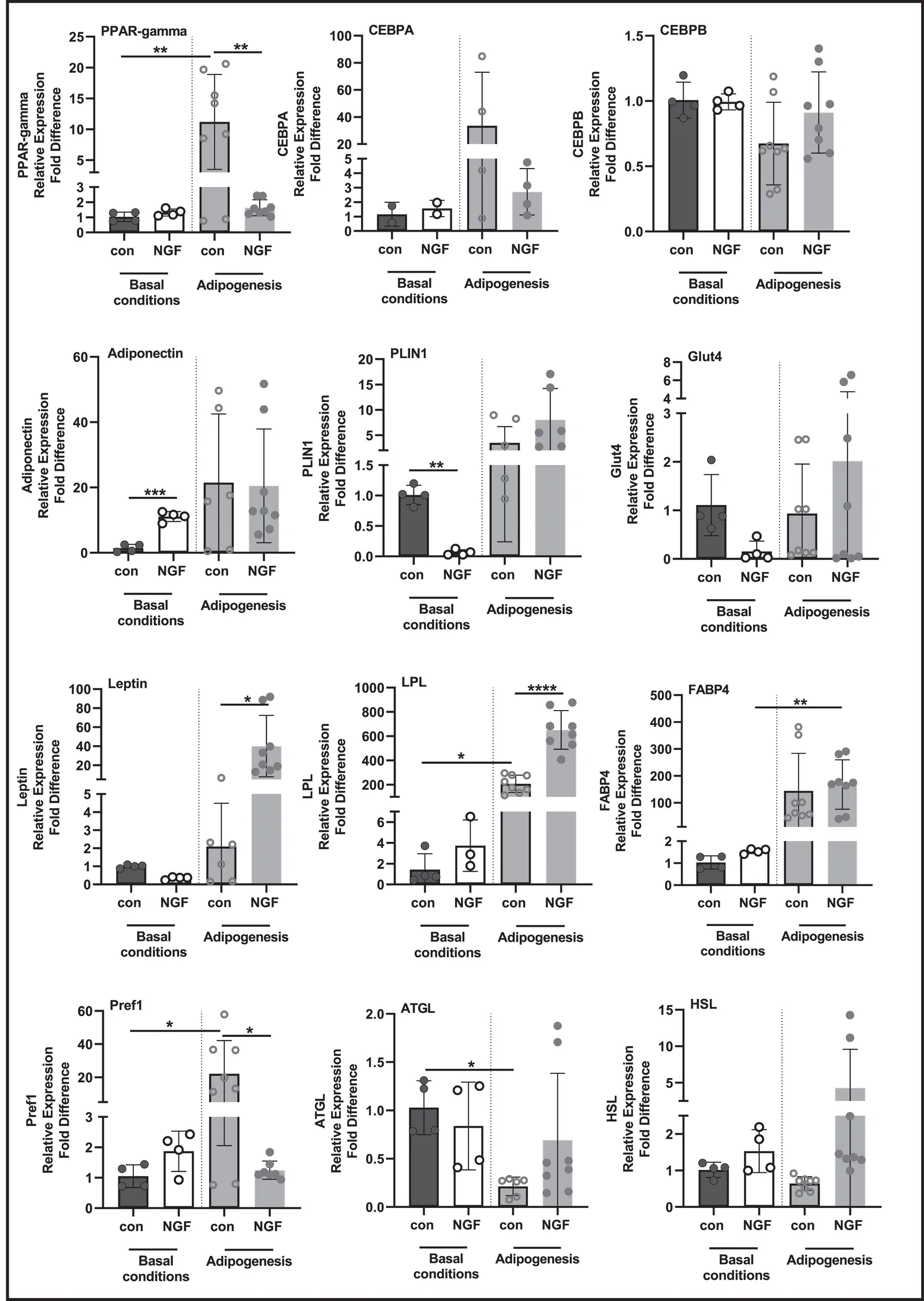
Effect of NGF on adipogenic markers during white adipogenesis of human pre-adipocytes: human pre-adipocytes were cultured in growth medium or adipogenic medium plus/minus NGF (100 ng/mL) for 15 days and measured the mRNA levels of adipogenic markers (PPAR-gamma, CEBPA, CEBPB, adiponectin, PLIN1, Glut4, leptin, LPL, FABP4, Pref1, HSL, and ATGL) by RT-PCR. GAPDH was used as a housekeeping gene. Data are depicted as mean ± SEM and represent the fold difference of the respective mRNA levels measured in control cells (cultured in growth media). Data from a human biopsy. *P < .05, **P < .01, ***P < .001, and ****P < .0001 denote statistical significance compared to the levels measured between indicated groups.

As NGF increased metabolism in our in vitro experiments in human white mature adipocytes and regulated lipolytic mechanisms in in vitro and in ex vivo experiments in human explants, we investigated whether NGF affected adipogenic genes during the in vitro differentiation of human pre-adipocytes to white adipocytes. Indeed, we cultured human pre-adipocytes with either growth medium or adipogenic medium plus/minus NGF (100 ng/mL) for 15 days and measured the mRNA levels of detected adipogenic markers (Fig. 5). The results showed that NGF increased adiponectin mRNA levels and at the same time decreased PLIN1 mRNA levels from human pre-adipocytes cultured in basal growth conditions for 15 days. During the differentiation process of human white adipocytes, NGF decreased PPAR-gamma and Pref1 mRNA levels while it increased leptin and LPL levels, all compared to the indicated mRNA levels measured in control cells.

### NGF increased both mitochondrial respiration and glycolysis rates in 3T3L1 pre-adipocytes while beta oxidation was not crucial for the basal ATP respiration process of 3T3L1 pre-adipocytes

**Figure 6:**
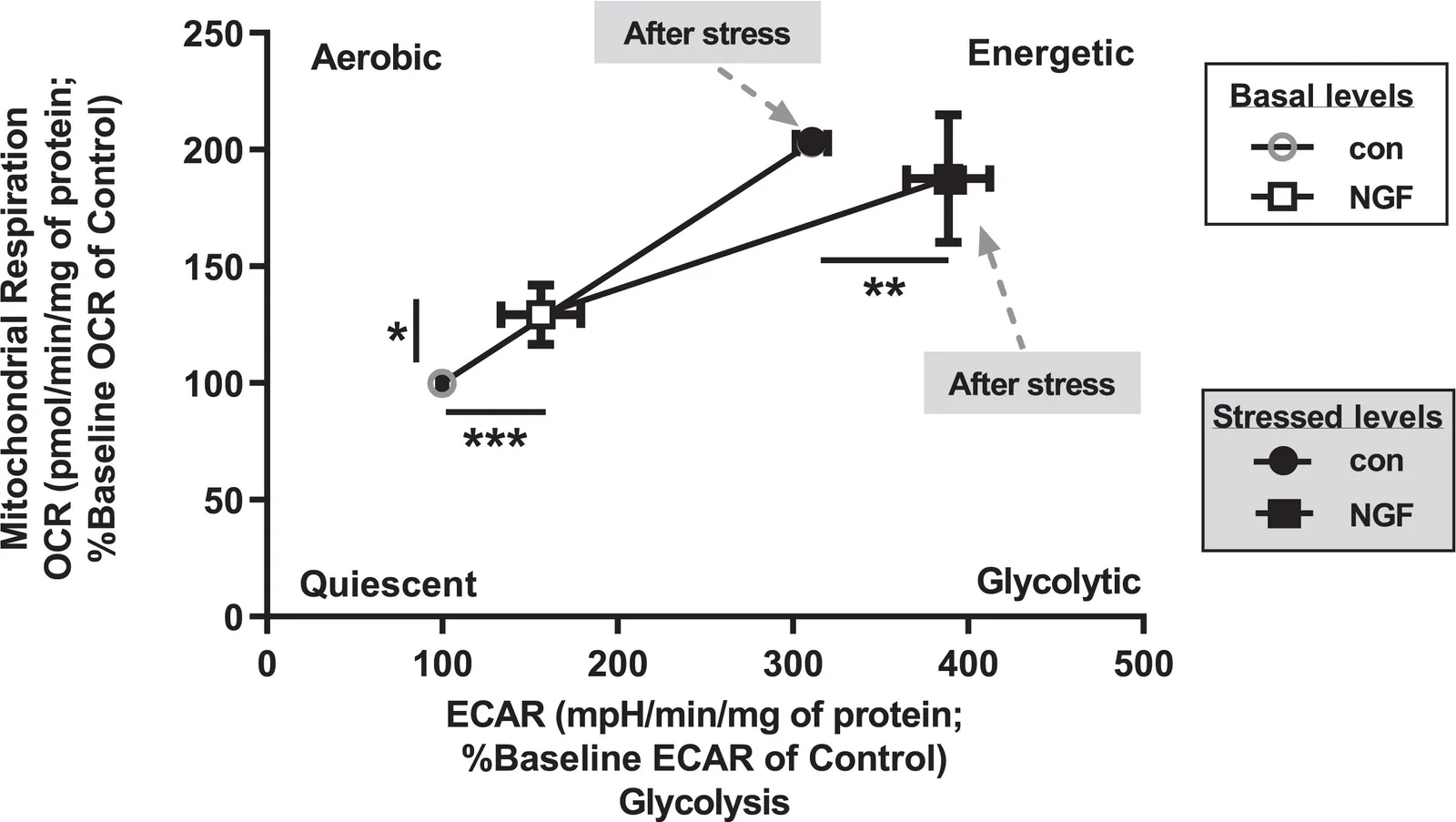
NGF increased the rates of both mitochondrial respiration and glycolysis in 3T3L1 pre-adipocytes. 3T3L1 pre-adipocytes were cultured in the presence or absence of NGF (100 ng/mL) and the basal levels of mitochondrial respiration (OCR-basal) and glycolysis (ECAR-basal) in real-time were measured during a 3.5-hour period by the Seahorse analyzer. Then, oligomycin and FCCP were simultaneously added to the cells, and the OCR-stressed levels and ECAR-stressed levels were uninterruptedly measured. The results are shown as mean ± SEM% and represent the percentage of baseline of the untreated group for either OCR or ECAR levels, respectively (*n* = 6-12 from a total of 6 experiments).*P < .05, **P < .01 and ***P < .001 denote statistical significance compared to the levels measured in the control group at basal or stressed points, respectively.

We next investigated whether NGF affects mitochondrial respiration and glycolysis rates in mouse 3T3L1 pre-adipocytes that express NGF and its receptors. According to our results, the 3.5-hour incubation of 3T3L1 pre-adipocytes with NGF (100 ng/mL) increased the baseline of both mitochondrial respiration (OCR) and glycolysis (ECAR) rate compared to the baseline of OCR of untreated group (mean ± SEM% OCR-baseline: NGF 129.3% ± 12.7% vs control 100.0% ± 1.5%, *P* < .05) and the baseline of ECAR of untreated group (ECAR-baseline: NGF 156.3% ± 22.8% vs control 100.0% ± 1.7%, *P* < .001), respectively. Moreover, when the cells were forced to use all their energy supplies by the simultaneous addition of the stressor compounds oligomycin (an inhibitor of ATP synthase) and FCCP (a mitochondrial uncoupling agent), the incubation with NGF (100 ng/mL) further significantly increased stressed-induced glycolysis rate in comparison with the stressed-glycolysis rate of the untreated group (ECAR-stressed levels: NGF 388.7% ± 23.7% vs control 310.8% ± 9.1%, *P* < .01) (Fig. 6). Subsequently, we used the above data and calculated the metabolic potential of 3T3L1 pre-adipocytes in response to NGF (described in “Methods”). As the metabolic potential illustrates the maximum ability of the cells for respiration or glycolysis, our data showed that the co-incubation of NGF with the TrkA inhibitor completely reversed both effects of NGF on mitochondrial respiration and glycolysis (Supplemental Fig. S4A (18)).

To elucidate the contribution of long-chain fatty acid oxidation to mitochondrial respiration of 3T3L1 pre-adipocytes we analyzed the effect of etomoxir, which inhibits the enzyme carnitine palmitoyl-transferase 1A (CPT1A) (24). The ATP respiration of cells were measured as the increase in OCR levels in Seahorse analyzer. We found that neither the acute exposure nor the pre-incubation for 60 minutes to etomoxir had any effect on the OCR levels that accounts for the ATP respiration process in 3T3L1 pre-adipocytes (Supplemental Fig. S4B (18)).

### The role of NGF on beta oxidation and glucose uptake in human pre-adipocytes

To test the contribution of NGF on long-chain fatty acids oxidation process in human pre-adipocytes, we co-exposed cells to etomoxir plus NGF. Etomoxir abolished acutely the ATP respiration process in human pre-adipocytes and the co-exposure of cells to etomoxir plus NGF marginally increased OCR levels compared to the OCR levels measured by the acute exposure to etomoxir alone (Supplemental Fig. S5A (18)). The effect of etomoxir on the inhibition of beta oxidation was reversible. Indeed, when cells were pre-exposed to etomoxir for 60 minutes, the OCR levels almost reached the OCR measurements of controls (cells not exposed to any substance) (Supplemental Fig. S5B (18)). Again, NGF did not significantly alter mitochondrial respiration upon the etomoxir-driven blockage in human pre-adipocytes.

To evaluate the effect of NGF on glucose uptake, we treated human pre-adipocytes isolated from SVF fraction with NGF and measured glucose uptake. Glucose uptake was not affected by NGF in pre-adipocytes (Supplemental Fig. S5B (18)).

### The effect of NGF in naïve and LPS-induced pro-inflammatory factors in primary human white mature adipocytes and human pre-adipocytes

We have previously shown that depending on stress signals, the responses of mouse and human white adipocytes and pre-adipocytes involve the production of inflammatory factors (15). For this purpose, we examined the pro-inflammatory profile of human white mature adipocytes, and our results showed that human white mature adipocytes secreted high levels of IL-8 and IL-6 and lower levels of TNFα and IL-1β (Table 1). Low secretion levels of IFNγ were detected in SVF cells but not in white adipocytes. IL-1β levels were detected in the supernatants of cultured white adipocytes but not in SVF cells. LPS was a potent inducer of the levels of pro-inflammatory cytokines both in primary human SVF cells and white mature adipocytes. It should be noted that the secretions of TNFα and IL-17α was undetected in cultured SVF cells and were only evident in SVF cells upon LPS stimulation. As the chemokine IL-8 and the cytokine IL-6 were secreted by both human white mature adipocytes and pre-adipocytes, we next analyzed the effects of NGF on the mRNA levels of those pro-inflammatory markers.

**Table 1:** 24-hour basal and LPS-induced secretion of pro-inflammatory cytokines in human SVF cells and white mature adipocytes

	IL-8	IL-6	TNFα	IL-1β	IFNγ	IL-17α
SVF cells						
Basal levels	225.6 ± 65.9	2289.0 ± 12.3	—	—	0.48 ± 0.31	—
LPS	3892 ± 341	2403.0 ± 24.0	34.9 ± 1.1	—	3.76 ± 0.68	1.3 ± 0.9
White mature adipocytes						
Basal levels	3849 ± 218	2217.0 ± 24.0	51.7 ± 1.5	4.9 ± 0.9	—	—
LPS	4177 ± 39	2350.0 ± 0.3	201.6 ± 8.7	6.5 ± 0.8	—	—

24-hour basal and LPS-induced secretion of IL-8, IL-6, TNFα, IL-1β, IFNγ, and IL-17α were measured by Luminex from human SVF cells and human white mature adipocytes.

Results are presented as MEAN ± SEM (pg/mL for all). (−) represents non-detected secretion levels.

Abbreviations: IFNγ, interferon gamma; IL-1β, interleukin 1 beta; IL-6, interleukin 6, IL-8, interleukin 8, IL-17α, interleukin 17 alpha, LPS, lipopolysaccharide (100 ng/mL); SVF, stromovascular fraction, TNFα, tumor necrosis factor alpha;

**Figure 7:**
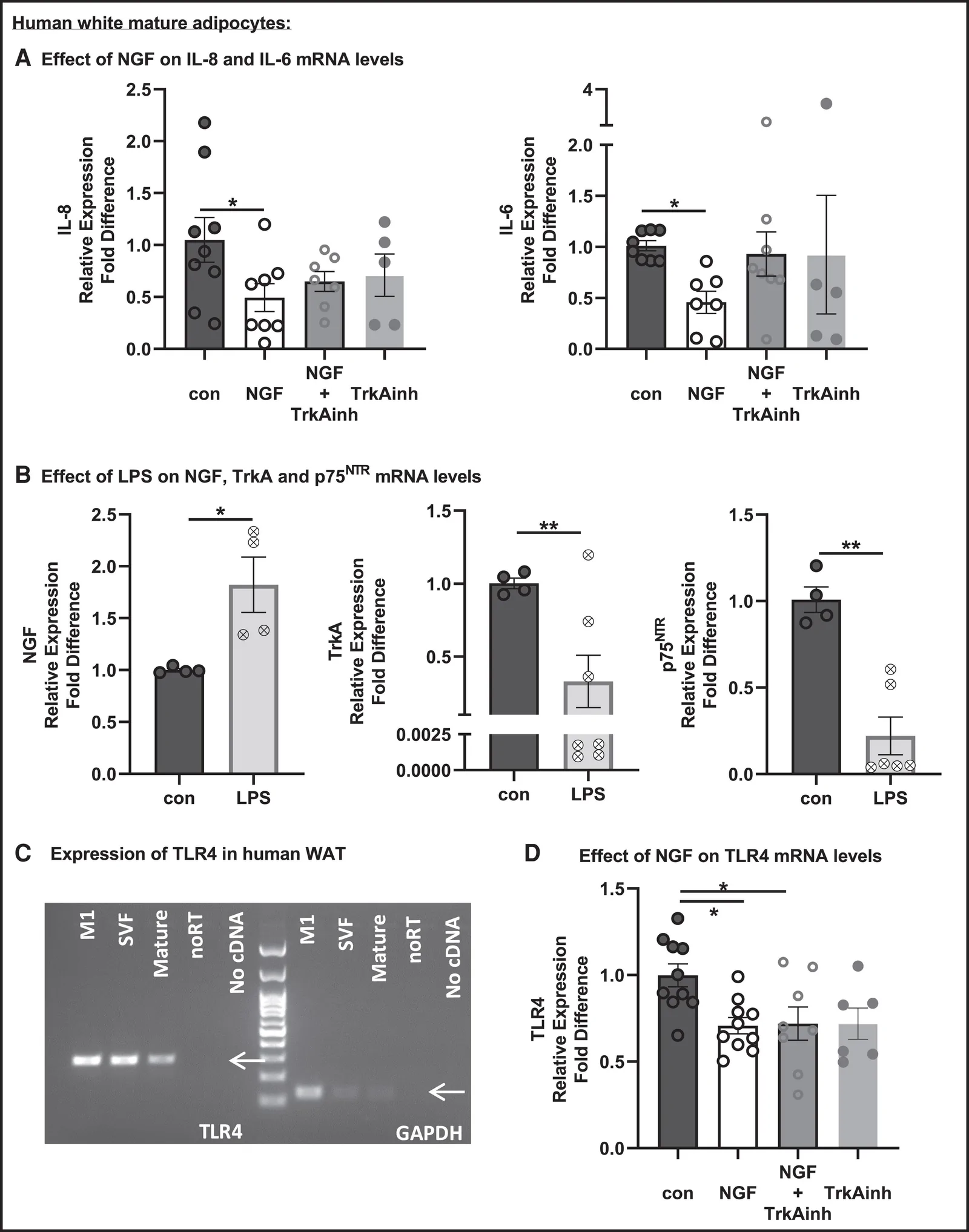
Effect of NGF on inflammatory factors and LPS on the expression levels of NGF and its receptors in human white mature adipocytes. (A) Human white mature adipocytes from 3 independent human biopsies were cultured in the presence of NGF (100 ng/mL) plus/minus TrkA inhibitor (10^−6^ M) for 24 hours and the mRNA of IL-8 or IL-6 were measured by RT-PCR. Data are presented as mean ± SEM and represent the fold difference of the respective mRNA levels measured in the control group. (B) Human white mature adipocytes were treated with LPS (100 ng/mL) for 24 hours and the mRNA levels of NGF, TrkA, and p75^NTR^ were measured by RT-PCR. Data are depicted as mean ± SEM and represent the fold difference of the respective mRNA levels measured in the control groups. Data show the results from cells taken from 2 independent human biopsies. *P < .05, and **P < .01 denote statistical significance of the respective levels compared to those levels measured in the control group. (C) The expression of TLR4 in human WAT. The mRNA levels of TLR4 measured by RT-PCR in human primary M1 macrophages, human SVF samples, and human primary white adipocytes (Mature). GAPDH was used as a housekeeping gene. Samples without reverse transcriptase during cDNA synthesis (noRT) and without cDNA during RT-PCR (No cDNA) were also shown. (D) Effect of NGF in the expression levels of TLR4 in human white adipocytes. Human white mature adipocytes were cultured in the presence of NGF (100 ng/mL) plus/minus TrkA inhibitor (10^−6^ M) for 24 hours and the mRNA of TLR4 was measured by RT-PCR. Data in graphs are presented as mean ± SEM and represent the fold difference of the mRNA levels measured in the control group. *P < .05. Data for TLR4 were collected from 2 different human biopsies.

We confirmed that a 24-hour incubation of human white mature adipocytes with NGF (100 ng/mL), in vitro, significantly downregulated the mRNA levels of IL-8 (mean ± SEM: NGF 0.49 ± 0.13 fold difference of IL-8 mRNA levels vs control 1.05 ± 0.21, *P* < .05) and IL-6 (NGF 0.46 ± 0.11 fold difference of IL-6 mRNA levels vs control 1.01 ± 0.05, *P* < .05) (Fig. 7A). It is noteworthy to mention that this action was partly mediated via the TrkA receptor, as co-exposure of NGF (100 ng/mL) and TrkA receptor inhibitor (10^−6^ M) reversed this effect in the case of IL-6 expression.

### Reciprocal regulation of receptor levels; the pro-inflammatory factor LPS downregulated the transcription of TrkA and p75^NTR^ while NGF downregulated the transcription of TLR4 in human white mature adipocytes

We next studied the effects of LPS on metabolic parameters and lipids storage in human white mature adipocytes. Indeed, a statistically significant reduction of the levels of mitochondrial mass was observed after 24 hours incubation with LPS (Supplemental Fig. 6A (18)), while this effect was diminished 30 hours post-treatment with LPS (data not shown). At the same time, LPS increased the amount of stored lipids and decreased the mRNA levels of PPAR-gamma and adiponectin all in human white mature adipocytes (Supplemental Fig. 6B (18)).

We further examined whether treatment with LPS could also affect the levels of NGF and its receptors and vice versa (if treatment with NGF affected the expression of receptor TLR4 in human white adipocytes). We found a transient decrease in the secretion of NGF 6 hours after exposure of human white adipocytes to LPS (100 ng/mL) (LPS 72.0 ± 4.3 pg/mL NGF vs control 100.0 ± 11.5, *P* < .05) measured by Luminex, which was later followed (at 24 hours) by an increase in the mRNA levels of NGF measured by RT-PCR (Fig. 7B). In addition, LPS robustly decreased the mRNA levels of both TrkA (LPS 0.33 ± 0.18-fold difference of TrkA mRNA levels vs control 1.00 ± 0.04, *P* < .01) and p75^NTR^ (LPS 0.22 ± 0.11-fold difference of p75^NTR^ mRNA levels vs control 1.22 ± 0.24, *P* < .01) in human white mature adipocytes (Fig. 7B).

LPS activates the M1 response in macrophages by binding to the receptor TLR4 (25) and we have previously shown that the receptor TLR4 is present in mouse 3T3L1 pre-adipocytes (15). Our present results reveal that the mRNA levels of TLR4 were strongly expressed in both human pre-adipocytes and human white mature adipocytes. Interestingly, the expression levels appeared to be comparable to those detected in isolated human M1 macrophages that were used as a positive control (Fig. 7C).

We next searched if NGF could affect the TLR4 mRNA levels in human white mature adipocytes. Indeed, 24-hour exposure of human white mature adipocytes to NGF (100 ng/mL) downregulated the mRNA levels of TLR4 (*P* < .05). This action, however, was not reversed by the TrkA receptor inhibitor (Fig. 7D). Remarkably, the exposure of human white mature adipocytes to TrkA inhibitor alone (10^−6^ M) did not have any significant effect on TLR4 mRNA levels (Fig. 7D).

### The receptors TrkA and p75^NTR^ are both expressed in 3T3L1 cells, and NGF downregulated CXCL1 secretion in 3T3L1 pre-adipocytes via TrkA

**Figure 8:**
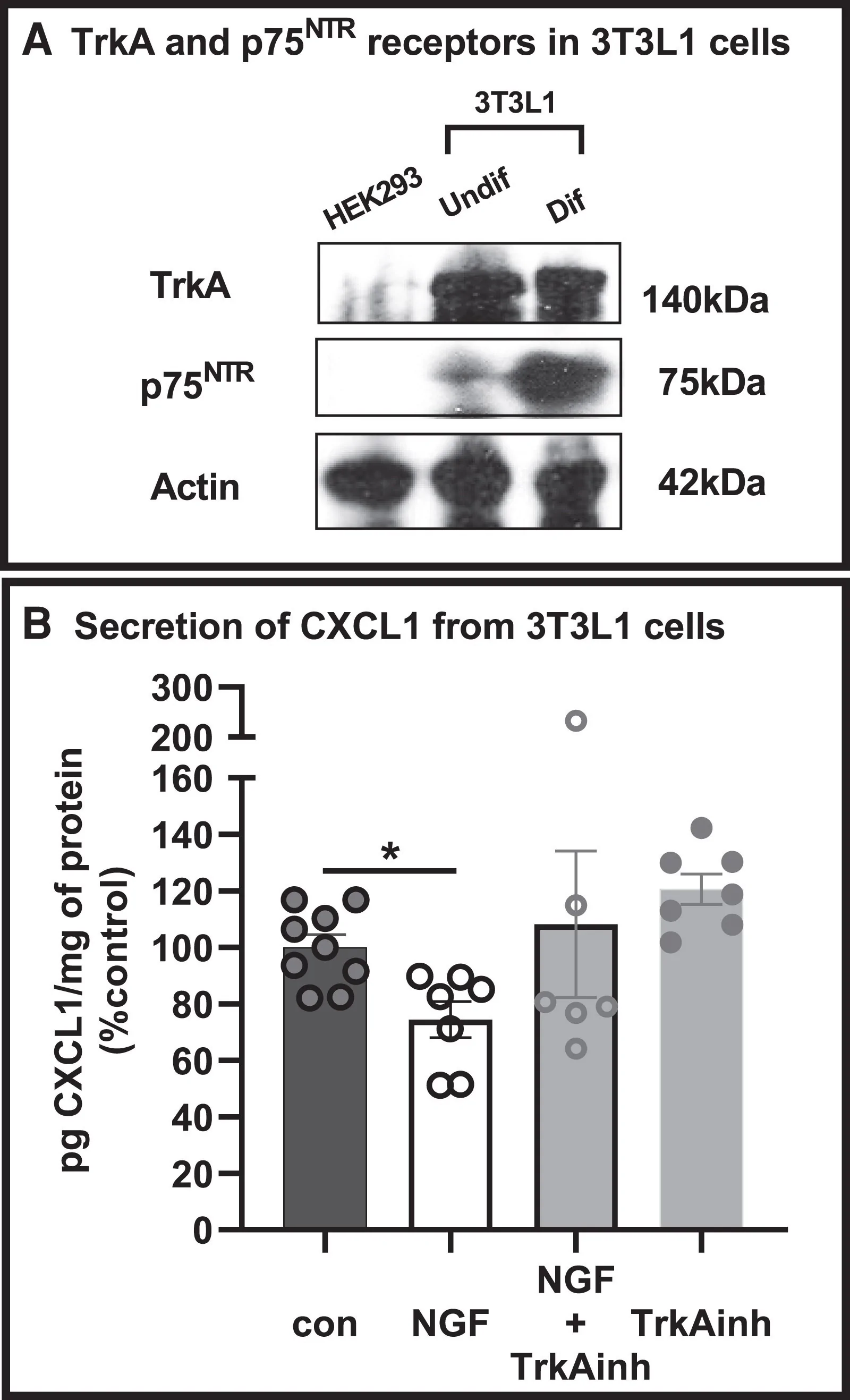
TrkA and p75^NTR^ are detected in 3T3L1 cells and NGF downregulated the secretion of CXCL1 in 3T3L1 pre-adipocytes via TrkA. (A) Protein levels of TrkA and p75^NTR^ receptors examined by Western blotting in mouse 3T3L1 pre-adipocytes (Undif) and in vitro differentiated-to-white 3T3L1 adipocytes (Dif). HEK293 cells were used as a negative control and actin was used as a housekeeping gene. (Β) Secretion levels of CXCL1 after 3 hours treatment of NGF (100 ng/mL) plus/minus TrkA inhibitor (10^−6^ M) measured by ELISA in 3T3L1 pre-adipocytes. Data are presented as the percentage of mean ± SEM of CXCL1 levels measured in the respective control groups and were collected from 2 independent experiments. *P < .05 represents statistically significant CXCL1 levels compared to the same levels measured in the control cells.

Our experimental data confirmed the existence of NGF receptors in 3T3L1 cells. Indeed, the TrkA receptor was detectable in both 3T3L1 pre-adipocytes and differentiated-to-white 3T3L1 adipocytes. Furthermore, the p75^NTR^ receptor protein levels were shown to be abundantly presented in differentiated-to-white 3T3L1 adipocytes compared to the p75^NTR^ protein levels detected in 3T3L1 pre-adipocytes (Fig. 8A).

IL-8 was the chemokine that was primarily secreted by human adipocytes (Table 1) and CXCL1 is the analogue of IL-8 in murine models (26). As NGF downregulated the mRNA levels of IL-8 in human adipocytes, we analyzed the effect of NGF on the secretion of CXCL1 in 3T3L1 pre-adipocytes. Our data showed that exposure of 3T3L1 pre-adipocytes to NGF (100 ng/mL) decreased CXCL1 secretion after 3 hours of treatment compared to control cells (*P* < .05). Interestingly, this effect was reversed by the TrkA inhibitor at 10^−6^ M (Fig. 8B), while exposure to the TrkA inhibitor alone marginally increased CXCL1 levels (120.6% ± 5.4% vs control 100.0% ± 4.5%, *P* = .0515) in 3T3L1 pre-adipocytes.

## Discussion

Our data confirmed the working hypothesis that NGF, and its receptors TrkA and p75^NTR^, are expressed in both human and mouse white adipocytes and pre-adipocytes. We also showed that NGF increased lipolysis in human WAT and in vitro exposure of human and mouse adipocytes to NGF increased their metabolic activity and their anti-inflammatory phenotype. Regarding the involvement of TrkA on the effects of NGF in the regulation of metabolic and inflammatory pathways, we found that the TrkA inhibitor partially abolished some of these effects, while the pro-inflammatory factor LPS decreased the levels of NGF receptors in human white adipocytes.

### Expression of NGF and its receptors in human adipocytes and pre-adipocytes

We have detected NGF secretion levels in human white adipocytes in vitro and the mRNA of NGF that were in comparable levels in both human white adipocytes and pre-adipocytes isolated from visceral WAT depots. These results are in accordance with previous findings showing that NGF was released by human visceral adipose tissue explants. Furthermore, it has been proposed that the amount of NGF released by adipocytes was approximately 72% of that released by non-fat cells (27). Our study also showed for the first time that the receptor p75^NTR^ was abundantly expressed in both isolated human white adipocytes and pre-adipocytes and the receptor TrkA was detectable in the same cell populations. A similar expression pattern of p75^NTR^ and TrkA mRNA levels was previously shown in mouse primary adipocytes (2). TrkA represents the high-affinity receptor for NGF, while p75^NTR^ is its low-affinity receptor. It has been suggested that the co-expression of the 2 genes (p75^NTR^ and TrkA) can potentially lead to greater responsiveness to NGF (10^−11^ M) as specific domains in p75^NTR^ interacts with TrkA, increasing the binding characteristics of the receptor-tyrosine kinase in co-transfected cell lines (28, 29). Here, we propose that TrkA regulates metabolic and inflammatory mechanisms in human white adipocytes as the TrkA inhibitor partially abolished some of the effects of NGF.

### NGF in metabolism of human white adipocytes and pre-adipocytes

NGF is an established trophic factor for neurons (30) and accumulating evidence so far has expanded our knowledge of its trophic effects in non-neuronal cell types like lymphocytes, pancreatic beta cells and muscle cells (7-9). Our newly presented data depict that NGF increased the metabolic activity of human pre-adipocytes and the mitochondrial respiration and glycolysis rates of mouse 3T3L1 pre-adipocytes. Cells were exposed to different concentrations of NGF (1-1000 ng/mL) and the concentration of 100 ng/mL was chosen as the optimum concentration for consistent results. This concentration of NGF has already been used in other studies to achieve stable activity of cells (31). We also provide evidence that NGF affected the metabolic phenotype of human white mature adipocytes. Our experiments revealed a dynamic decrease in the amount of mitochondrial mass of human white enlarged adipocytes at 24 hours of exposure to NGF. The loss of the amount of mitochondrial mass in human white mature adipocytes was not attributed to cell death, as our parallel experiments detecting their metabolic activity by MTT demonstrated that cells exposed to NGF for 24 hours were marginally more metabolically energetic than controls (cells not exposed to NGF). We hypothesize that this effect could be attributed to a rescue mechanism for an essential reorganization of white enlarged adipocytes, as has already been proposed, in both neurogenesis and nerve regeneration by NGF (32). Along with the reduction of mitochondrial mass, NGF upregulated the mRNA of PPAR-gamma, CEBPA, and adiponectin (at 24 hours) in human white mature adipocytes. PPAR-gamma is an important nuclear receptor responsible for mitochondria biogenesis (32) and CEBPA acts in conjunction with PPAR-gamma to promote adipogenesis (33, 34). Furthermore, a recent study showed that the intervention with adiponectin in renal tubular epithelial cells increased their mitochondrial mass, and the authors proposed that adiponectin promoted repair of cells by regulating mitochondrial biogenesis and function (35). Therefore, our hypothesis that NGF coordinated mitochondrial remodeling in human white enlarged adipocytes is strengthened. We propose that the mitochondrial remodeling of white enlarged adipocytes together with the upregulation of adipogenic factors by NGF could contribute to a beneficial metabolic phenotype of white adipocytes thus, contributing to the overall WAT plasticity in humans.

### Fatty acids and glucose as energy sources in pre-adipocytes

To elucidate the contribution of long-chain fatty acid oxidation to mitochondrial respiration of pre-adipocytes we analyzed the effect of etomoxir, which inhibits the enzyme CPT1A (24). The acute exposure to etomoxir abolished the ATP respiration process in human pre-adipocytes but not in 3T3L1 pre-adipocytes. We concluded that fatty acids are used as energy source by human pre-adipocytes as etomoxir completely downregulated the ATP respiration process. When human pre-adipocytes were pre-exposed to etomoxir for 60 minutes, ATP respiration returned to almost the same levels as measured in control cells (without exposure to etomoxir). Therefore, it appeared that human pre-adipocytes regained their ability to increase their mitochondrial respiration by alternative pathways when the beta oxidation process was not available.

In another set of experiments, we measured the mRNA levels of Glut4, which is the predominant glucose transporter used by adipocytes (36). We measured higher expression pattern of Glut4 in primary human white adipocytes (40-fold) compared to that measured in primary human pre-adipocytes. Chronic exposure to NGF decreased the mRNA levels of Glut4 in human pre-adipocytes. The activation of glucose transport in the cells is a dynamic process that depends on the preexisting levels of Glut4 that are translocated to the plasma membrane in response to insulin. It has been proposed that NGF induces glucose-dependent insulin secretion in rat pancreatic beta cells (6) and that tissue-specific deletion of NGF or TrkA or acute disruption of TrkA signaling, impairs glucose tolerance and insulin secretion in mice (37). Our experiments in human pre-adipocytes revealed that NGF did not affect glucose uptake in human pre-adipocytes despite the reduction of Glut4. These results suggest that there is no direct effect of NGF in fatty acid oxidation or glucose uptake.

### NGF and lipogenesis

While our findings suggested that NGF did not affect glucose uptake or beta oxidation in human pre-adipocytes, NGF maintained their pre-adipocyte phenotype by the differential regulation of transcription factors and adipokines. Indeed, the chronic exposure of NGF during the white adipogenesis process of human pre-adipocytes, did not affect their differentiation per se but decreased PPAR-gamma mRNA levels. Furthermore, chronic exposure of human pre-adipocytes to NGF supported their pre-adipocyte phenotype by promoting adiponectin and decreasing PLIN1 levels. PPAR-gamma is an insulin-sensitizing nuclear receptor predominantly expressed in adipocytes (38) that alters the transcription of genes involved in the regulation of glucose and lipid metabolism (39, 40). Furthermore, decreasing levels of adiponectin in obesity have been linked to the development of diabetes. The fact that NGF has homology with pro-insulin (41) and that serum levels of NGF are decreased in patients with diabetes mellitus (42) could suggest that NGF might participate in alternative metabolic mechanisms controlling glucose sensitivity. Our results further confirmed the anti-adipogenic properties of NGF in human pre-adipocytes by decreasing lipid accumulation in in vitro culture of human white mature adipocytes and increasing lipolysis in ex vivo WAT tissue explants. In support of our findings, it has been suggested that the denervation of mouse WAT (using a viral transneuronal tract tracer, the pseudorabies virus) increased WAT mass and WAT fat cell number in both inguinal WAT and retroperitoneal WAT (43).

### The anti-inflammatory effect of NGF in adipocytes

We first searched for the pro-inflammatory profile of human white mature adipocytes, and our results showed that human white mature adipocytes secreted high levels of IL-8 and IL-6 and lower levels of TNFα and IL-1β. We then analyzed the effects of NGF on the mRNA levels of IL-8 and IL-6 in human white adipocytes. We report that in vitro exposure of human white mature adipocytes to NGF inhibited the levels of IL-6 and IL-8 as well as the levels of the receptor TLR4. We also showed that TrkA is involved in NGF suppression of CXCL1 secretion (the analog of IL-8 in murine models (26)) since the TrkA inhibitor reversed this effect in 3T3L1 pre-adipocytes. Our findings regarding the anti-inflammatory effect of NGF in human white mature adipocytes and in 3T3L1 cells are in accordance with previous findings in human TLR-activated monocytes, where NGF reduces the inflammatory cytokine production (IL-1β, TNFα, IL-6, and IL-8) and induces the release of anti-inflammatory mediators (IL-10 and IL-1 receptor antagonist (44)). The authors suggested that when TrkA expression is defective, pro-inflammatory mechanisms that contribute to chronic tissue inflammation and damage are present. As we showed, the expression levels of TrkA in human white adipocytes are functional as the TrkA inhibitor partially abolished some of the effects of NGF in metabolic and anti-inflammatory processes in human white adipocytes. Moreover, as the activation of PPAR-gamma has also been suggested to down-regulate NFkB signaling (45), there could be another indirect mechanism of NGF via upregulation of PPAR-gamma to inhibit cytokines and TLR4 levels in human white adipocytes. Overall, our findings of the in vitro anti-inflammatory effects of NGF in adipocytes could support the findings by others that there are low serum NGF levels in patients with cardiometabolic-related diseases (atherosclerosis, obesity, type 2 diabetes, metabolic syndrome (5)) where systemic low-grade metabolic inflammation is also present. In accordance with that, Isakson and colleagues suggested that in hypertrophic adipose tissue the ability of pre-adipocytes to proliferate and differentiate is diminished, resulting in increased lipid intake from existing adipocytes and increased pro-inflammatory response (46).

### The LPS-driven decrease of the levels of NGF receptors in human white adipocytes

In our study, LPS was a potent activator of the inflammatory response in both human white mature adipocytes and SVF cells. Furthermore, in vitro exposure of human white adipocytes to LPS reduced the mRNA levels of TrkA and p75^NTR^. Similarly, it has been previously shown that IL-6 reduces the levels of NGF in adipocytes differentiated from 3T3L1 (11). As the levels of LPS are generally increased in obesity, and the TLR4 binding to pro-inflammatory stimuli like LPS regulates cytokine production (25), our results showed that the LPS-driven downregulation of NGF receptors may serve as an alternative pathway to maintain the low-grade inflammatory status of human white enlarged adipocytes in obesity.

### Limitations of the study

Human primary SVF cells were isolated from visceral WAT depots and are composed of many cell types including macrophages, pre-adipocytes, and other stromal cells. The phenotypic signature of those cells revealed the expression of markers of adipocytes; transcription factors (PPAR-gamma, CEBPA, CEBPB), adipokines (leptin, adiponectin), lipid metabolism markers (LPL, PLIN1), lipolytic enzymes (HSL, ATGL), glucose transporter protein in adipocytes (Glut4), all expressed in low levels except for the high expression levels of the pre-adipocyte marker, Pref1. Thus, we conclude that the SVF cells are composed mainly of pre-adipocytes, although the existence of small populations of other cell types cannot be totally excluded. To check the validity of our findings in human SVF pre-adipocytes, we also used the pre-adipocytes 3T3L1 cell line.

In conclusion, our results confirmed the presence of NGF and its receptors in adipocytes freshly isolated from human abdominal WAT, the role of NGF as a metabolic and inflammatory regulator in adipocytes and that the NGF system is under the control of LPS that is enhanced in obesity in order to retain low-grade metabolic inflammation and metabolic dysregulation within the WAT.

## Data Availability

Some or all datasets generated during and/or analyzed during the current study are not publicly available but are available from the corresponding author on reasonable request.

## Abbreviations

ATGL: adipose triglyceride lipase
BAT: brown adipose tissue
BSA: bovine serum albumin
CEBPA: CCAAT/enhancer binding protein alpha
CPT1A: carnitine palmitoyl-transferase 1A
CXCL1: C-X-C motif chemokine ligand 1
ECAR: extracellular acidification rate
FCCP: carbonyl cyanide 4-(trifluoromethoxy)phenylhydrazone
Glut4: glucose transporter 4
HSL: hormone-sensitive lipase
IFNγ: interferon gamma
IL-: interleukin
LPS: lipopolysaccharide
MTT: 3-(4,5-dimethylthiazolyl-2)-2,5-diphenyltetrazolium bromide
NGF: nerve growth factor
OCR: oxygen consumption rate
p75^NTR^: pan-neurotrophin receptor
PLIN1: perilipin1
PPAR: peroxisome proliferator-activated receptor
Pref1: pre-adipocyte marker
RT-PCR: reverse transcription–polymerase chain reaction
SEM: standard error of the mean
SVF: stromal vascular fraction
TNFa: tumor necrosis factor alpha
TLR4: toll-like receptor 4
TrkA: tropomyosin-related kinase A
WAT: white adipose tissue
